# Effect of Illumination on Ocular Status Modifications Induced by Short-Term 3D TV Viewing

**DOI:** 10.1155/2017/1432037

**Published:** 2017-02-27

**Authors:** Yuanyuan Chen, Yuwen Wang, Xinping Yu, Aiqin Xu, Jian Jiang, Hao Chen

**Affiliations:** The Affiliated Eye Hospital of Wenzhou Medical University, Wenzhou, Zhejiang 325000, China

## Abstract

*Objectives.* This study aimed to compare changes in ocular status after 3D TV viewing under three modes of illumination and thereby identify optimal illumination for 3D TV viewing.* Methods.* The following measures of ocular status were assessed: the accommodative response, accommodative microfluctuation, accommodative facility, relative accommodation, gradient accommodative convergence/accommodation (AC/A) ratio, phoria, and fusional vergence. The observers watched 3D television for 90 minutes through 3D shutter glasses under three illumination modes: A, complete darkness; B, back illumination (50 lx); and C, front illumination (130 lx). The ocular status of the observers was assessed both before and after the viewing.* Results.* After 3D TV viewing, the accommodative response and accommodative microfluctuation were significantly changed under illumination Modes A and B. The near positive fusional vergence decreased significantly after the 90-minute 3D viewing session under each illumination mode, and this effect was not significantly different among the three modes.* Conclusions.* Short-term 3D viewing modified the ocular status of adults. The least amount of such change occurred with front illumination, suggesting that this type of illumination is an appropriate mode for 3D shutter TV viewing.

## 1. Introduction

3D TV and stereo video games have become increasingly popular in recent years. When viewing 3D TV, slightly different images with different extents of offset are dichoptically presented to both eyes and fused into one single image with depth perception.

Despite improvements in modern 3D viewing techniques, several studies have shown that prolonged exposure to 3D TV can temporally modify ocular status [1], including the conflict between accommodation and convergence while viewing stereo displays [2], a reduction in accommodation velocity [3], transient myopia [4], a reduction in pupillary movement in the near reflex [5], and microfluctuation in human adults [6], indicating some degree of visual plasticity in adults. Such ocular modifications induced by 3D TV have been suspected as a primary cause of visual discomfort [2, 7, 8].

Alternatively, there is evidence that inappropriate illumination may also cause ocular discomfort [9–11]. Appropriate illumination and image brightness of visual display terminals (VDT) may decrease discomfort from the flicker of the display [12]. However, it is unclear which commonly used illumination mode leads to the least amount of ocular status modulation when watching a 3D display in a living room. To answer this question, we assessed ocular status before and after a short-term (90 minutes) 3D viewing session with shutter glasses under three commonly used illumination modes [13, 14]: (A) complete darkness; (B) back illumination (50 lx); and (C) front illumination (130 lx). We show that a part of ocular status was significantly affected under all three illumination modes. Overall, the lowest degree of ocular modulation occurred with front illumination.

## 2. Materials and Methods

### 2.1. Participants

Thirty-two normal adults (16 females and 16 males; mean age: 23.63 ± 1.58 years) participated in the study. All the viewing observers had no history of ocular disease and had normal or corrected-to-normal visual acuity (see Table 1 for a summary of their characteristics). Informed consent was obtained from all the observers after an explanation of the nature and possible risks of the study. The study followed the tenets of the Declaration of Helsinki and was approved by the Ethics Committee of the Eye Hospital of Wenzhou Medical University.

### 2.2. Illumination Mode

**Figure 1:**
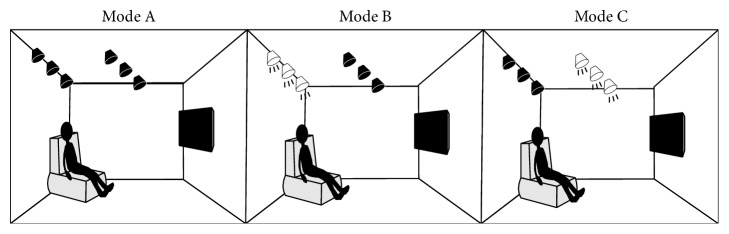
Illustration of our experimental environment for three illumination modes.

The study was conducted in an ordinary living room with white painted walls (7.5 × 3.3 m and 2.7 m high; see Figure 1 for an illustration). Observers were instructed to sit on a sofa that was located 2.5 m away from the television and watch a 3D movie using shutter glasses. Six 15-watt fluorescent lamps were mounted onto the ceiling. Each lamp could be controlled separately. Three commonly used illumination modes were studied in this experiment:* Mode A*,* Complete Darkness*, in which observers watched 3D television in complete darkness, without any source of illumination other than the television;* Mode B*,* Back Illumination*, in which only the three fluorescent lamps mounted onto the ceiling behind the viewers were turned on, and the luminance measured at the viewer's position was 50 lx; and* Mode C*,* Front Illumination*, in which only the three fluorescent lamps mounted onto the middle of the ceiling were turned on, and the luminance measured at the viewer's position was 130 lx.

### 2.3. Display

All visual stimuli were presented on a Samsung 3D television (Model number UA46D6000SJ, South Korea). The viewers looked at the screen through commercially available active shutter glasses, which were provided by Samsung for 3D viewing. The television had a screen size of 46 in, a screen resolution of 1920 × 1080 pixels, and a refresh rate of 200 HZ. The default settings of the television were as follows: back light 20, contrast gradient 100, brightness 100 lumens, clarity 59, chromaticity 50, and tone green 50/red 50, which resulted in a luminance of 750 cd/m^2^ on the screen and 23 cd/m^2^ on the glasses at a distance of 2.5 m.

### 2.4. Measurements

Ocular statuses, including accommodative response, accommodative microfluctuation, accommodative facility, positive and negative relative accommodation, gradient accommodative convergence/accommodation (AC/A), distant and near phoria, and positive and negative fusional vergence, were measured. These binocular visual function parameters were focused on in this study because these parameters have been widely used in previous studies of 3D TV viewing-induced visual discomfort [6, 15, 16].

The accommodative response and accommodative microfluctuation are sensitive indicators of the accommodation function. Accommodative microfluctuation represents the viability of the accommodative response and is the average amount of fluctuation in diopters around the mean accommodative response for a specified period  [17]. These parameters were measured at 25 cm, 40 cm, and 6 m with an infrared autorefractor (Grand Seiko WAM-5500 autorefractor, Grand Seiko, Co., Japan) at a rate of 5 times per second [18–20].

Positive and negative relative accommodation, AC/A, and positive and negative fusional vergence were measured using a phoropter (Topcon Model IS-600, Japan). Phoria was measured at 33 cm and 6 m using the von Graefe test (with a 3-measurement average). Accommodative facility was measured with a ±2.00 diopter flipper.

### 2.5. Procedure

To save time and reduce the potential discomfort from ocular examinations of the observers, we assessed all the ocular status measures prior to the start of the experiment, except for the accommodative response and accommodative microfluctuation. We set these measures as previewing baselines for all three illumination modes. This arrangement also made sense according to our pilot study, in which we asked five normal healthy adults to view 3D TV on different days and assessed their ocular status before 3D viewing for each day; we found that all of the above-mentioned measures of ocular status, except for the accommodative response and accommodative microfluctuation, were stable across different time points. Since the accommodative response and accommodative microfluctuation were different with each previewing test, we measured these parameters both before and after the 3D viewing with the three illumination modes.

**Figure 2:**
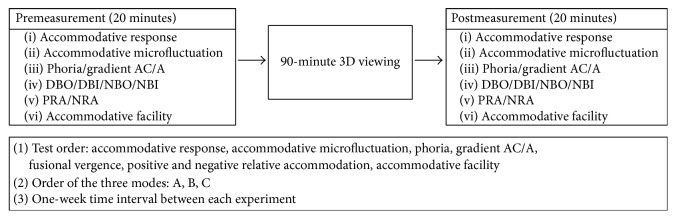
Illustration of our experimental sequence.

For each illumination mode session, we conducted the study in the following sequence: a premeasurement of the accommodative response and accommodative microfluctuation of the observers; a 20-minute break; a 90-minute 3D TV viewing session; and postmeasurement of the ocular status of each observer (including the accommodative response and accommodative microfluctuation as well as all the other measures mentioned above), which were measured immediately after the observers finished the 3D TV viewing session. The postmeasurement session was completed within 20 minutes for each individual. We assessed the different measures of ocular status in a fixed order in each session as follows: the accommodative response, accommodative microfluctuation, phoria, gradient AC/A, fusional vergence, relative accommodation, and then accommodative facility (Figure 2).

Three 3D movie VCDs were randomly selected for each observer, who was instructed to view the three movies under the three different illumination modes over three weeks. During the entire experiment, the TV was used with the same default settings, including brightness, contrast, color saturation, and clarity. Any other 3D display viewing was forbidden during this 3-week experimental period.

### 2.6. Statistical Analysis

Pre- and post-3D viewing measures were compared using a 2-tailed paired-samples* t*-test for each mode. The results from the three different illumination modes were compared using a three-way repeated measures ANOVA or one-way repeated measures ANOVA (*α* = 0.05). All the statistical analyses were performed using the IBM SPSS version 19.0 software, SPSS (Chicago, IL, USA).

## 3. Results

### 3.1. Accommodative Response

**Figure 3:**
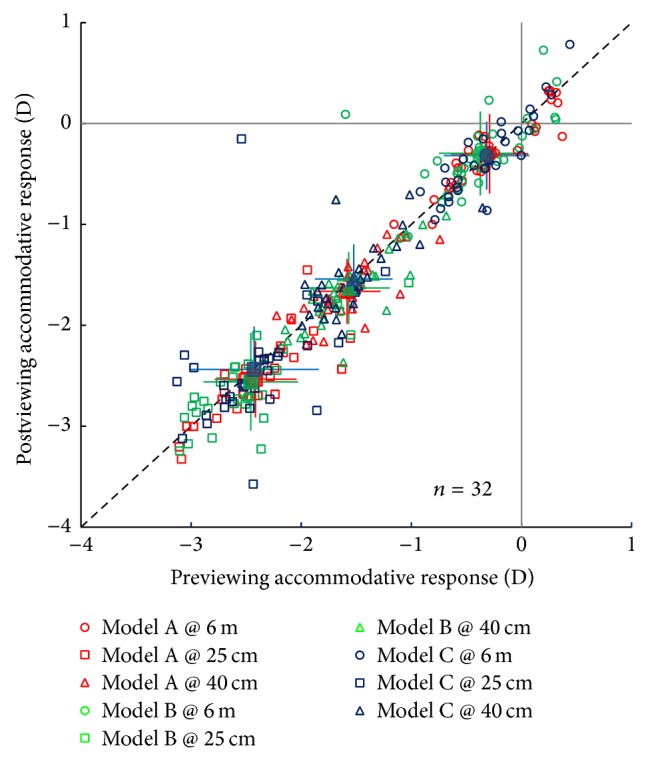
The effect of a 90-minute 3D viewing session on accommodative response. The postviewing accommodative responses of individuals are plotted as open points as a function of their previewing accommodative responses for the three illumination modes, which were measured at distances of 25 cm, 40 cm, and 6 m. The average values of the accommodative responses are plotted as solid points (the mean ± SD). The dashed line is the identity line (*X* = *Y*).

The accommodative responses at measuring distances of 25 cm, 40 cm, and 6 m before and after 3D viewing are plotted in Figure 3 for the three illumination modes. According to Figure 3, a 90-minute 3D viewing session did not appear to greatly alter the accommodative response for the conditions we measured. A repeated measures ANOVA for each illumination mode, with the measuring distance (i.e., 25 cm, 40 cm and 6 m) and test session (pre- and post-3D viewing) as within observers factors, indicated that the accommodative response was significantly changed after 3D viewing under Mode A (*F*(1,31) = 11.029, *p* = 0.002) but not under Mode B (*F*(1,31) = 0.661, *p* = 0.422) or Mode C (*F*(1,31) = 0.044, *p* = 0.836). A further 3-way ANOVA combining the results of Mode B and Mode C revealed that the effect of 3D viewing on the accommodative response was not significantly different between Mode B and Mode C; the joint effects of test session and mode were not significant, *F*(1,31) = 0.136, *p* = 0.715. These results indicate that, regarding the accommodative response, Mode B and Mode C are superior to Mode A.

### 3.2. Accommodative Microfluctuation

**Figure 4:**
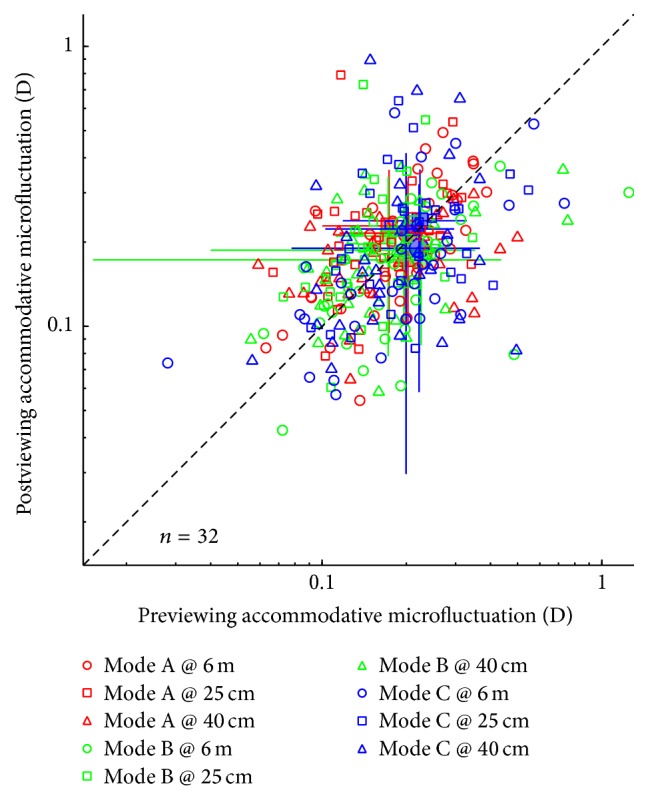
The effect of a 90-minute 3D viewing session on accommodative microfluctuation. The postviewing accommodative microfluctuations of individuals are plotted as open points as a function of their previewing accommodative microfluctuation measurements for the three illumination modes, which were measured at distances of 25 cm, 40 cm, and 6 m. The average values of the accommodative responses are plotted as solid points (the mean ± SD). The dashed line is the identity line (*X* = *Y*).

In Figure 4, the accommodative microfluctuation at measuring distances of 25 cm, 40 cm, and 6 m before and after 3D viewing is plotted for the three illumination modes. A repeated measures ANOVA for each illumination mode, with measuring distance (i.e., 25 cm, 40 cm, and 6 m) and test session (pre- and post-3D viewing) as within observers factors, showed that the accommodative microfluctuation was significantly changed after 3D viewing under Mode A (*F*(1,31) = 5.237, *p* = 0.011) and Mode B (*F*(1,31) = 4.233, *p* = 0.048) but not under Mode C (*F*(1,31) = 1.028, *p* = 0.360). The accommodative microfluctuation displayed a trend toward an increase after 90 minutes of 3D viewing under Mode A and under Mode B at a near distance. These results indicated that the accommodative microfluctuation was most stable under Mode C.

### 3.3. Other Accommodative Statuses

#### 3.3.1. Accommodative Facility

**Figure 5:**
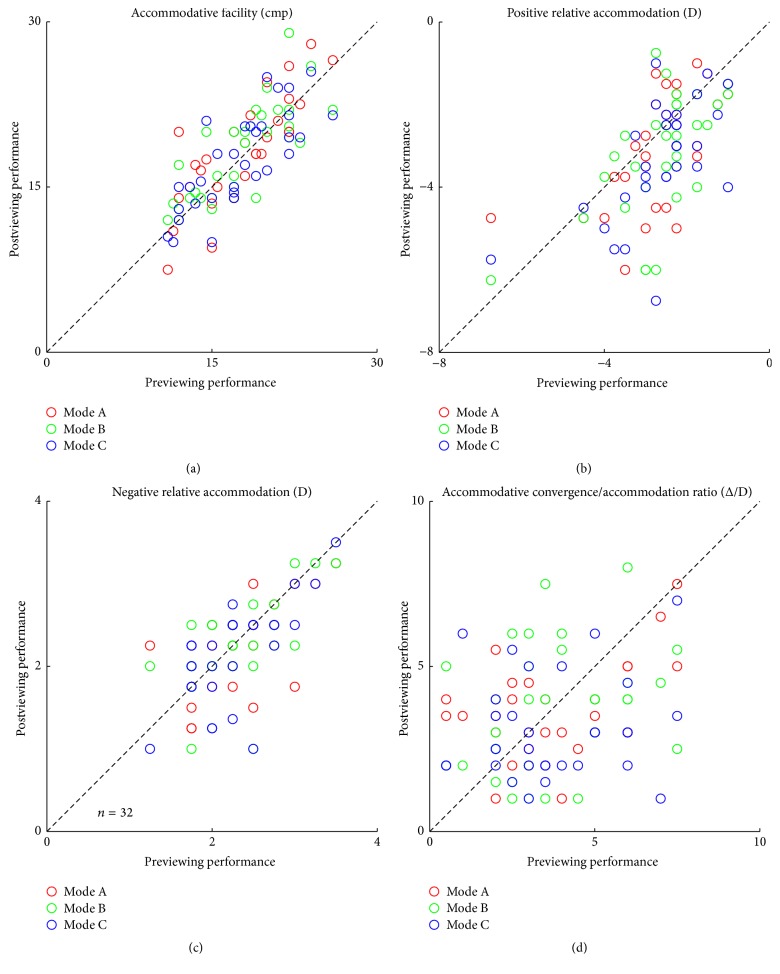
The effect of a 90-minute 3D viewing session on other accommodative statuses. The effect of a 90-minute 3D viewing session on accommodative facility (a), positive relative accommodation (b), negative relative accommodation (c), and the accommodative convergence/accommodation (AC/A) ratio (d). An identity line (*X* = *Y*) is plotted in each panel.

A two-tailed paired-samples* t*-test was conducted for the different illumination modes, which indicated no significant change in accommodative facility (*p* > 0.05) with any of the three modes. A one-way ANOVA also showed that there was no significant difference among the three modes (*F*(2,93) = 0.166, *p* = 0.847; Figure 5(a)).

#### 3.3.2. Positive Relative Accommodation (PRA)

The positive relative accommodation of the observers increased slightly after 3D viewing; however, this increase was not significant (2-tailed paired-samples* t*-test, *p* > 0.05). The results of the three modes were also not significantly different (*F*(2,93) = 0.416, *p* = 0.661; Figure 5(b)).

#### 3.3.3. Negative Relative Accommodation (NRA)

The negative relative accommodation of the observers decreased slightly after 3D viewing; however, this decrease was not significant (2-tailed paired-samples* t*-test, *p* > 0.05). The results of the three modes were also not significantly different (*F*(2,93) = 0.112, *p* = 0.894; Figure 5(c)).

#### 3.3.4. Accommodative Convergence/Accommodation Ratio (AC/A)

A 90-minute 3D viewing session did not considerably alter the AC/A (2-tailed paired-samples* t*-test, *p* > 0.05), and no significant differences were indicated among the three modes (*F*(2,93) = 1.138, *p* = 0.325; Figure 5(d)).

### 3.4. Phoria

**Figure 6:**
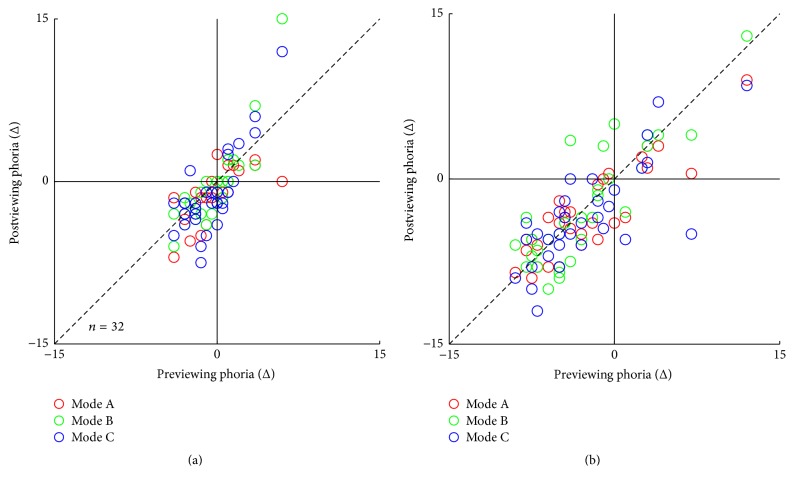
The effect of a 90-minute 3D viewing session on phoria. The effects of a 90-minute 3D viewing session on distance phoria (a) and near phoria (b). An identity line is plotted in each panel (*X* = *Y*).

The phoria before and after a 90-minute 3D viewing session for a given measuring distance (distant: 5 m; near: 40 cm) is plotted in Figure 6 for the three illumination modes. Before 3D TV viewing, 17 observers had exophoria, while 11 observers had esophoria, and the other 4 observers were orthophoric. After 3D TV viewing, both near and distance phoria had a tendency to show more exophoria, although there was no significant difference between the phoria data before and after a 90-minute 3D viewing session (*p* > 0.05). In addition, the phoria was not significantly different among the three modes of illumination (distance: *F*(2,93) = 0.442, *p* = 0.644; Figure 5(a); near: *F*(2,93) = 0.300, *p* = 0.741; Figure 6(b)).

### 3.5. Fusional Vergence

#### 3.5.1. Negative Fusional Vergence (BI)

**Figure 7:**
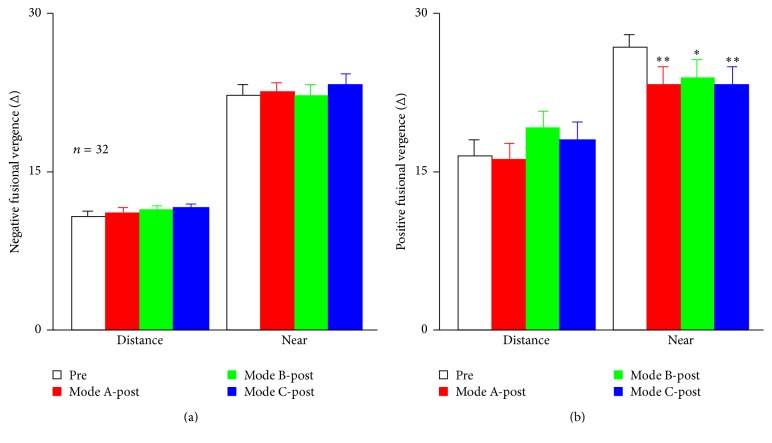
The effect of a 90-minute 3D viewing session on fusional vergence. The effect of a 90-minute 3D viewing on negative fusional vergence (a) and positive fusional vergence (b), which were measured at distant and near measuring conditions. Error bars indicate the standard error across 32 observers; ^*∗*^*p* < 0.05; 0.05 < ^*∗∗*^*p* < 0.01.

A 2-tailed paired-samples* t*-test showed that both near and distance negative fusional vergence were not significantly affected by the 3D viewing (*p* > 0.05); neither near nor distance negative fusional vergence was significantly different among the three modes (distance, *F*(3,124) = 0.568, *p* = 0.637; near, *F*(3,124) = 1.060, *p* = 0.369; Figure 7(a)).

#### 3.5.2. Positive Fusional Vergence (BO)

The near positive fusional vergence was significantly decreased after the 3D viewing (Mode A: *p* = 0.006; Mode B: *p* = 0.034; Mode C: *p* = 0.005). Neither the near nor distance positive fusional vergence was significantly different among the three modes (near: *F*(2,93) = 0.19, *p* = 0.982; distance: *F*(2,93) = 0.842, *p* = 0.473; Figure 7(b)).

*In summary*, after 3D TV viewing, the accommodative response was significantly changed under illumination Modes A, while accommodative microfluctuation was significantly changed under illumination Modes A and B; the near positive fusional vergence decreased significantly after a 90-minute 3D viewing session under each illumination mode, and this effect was not significantly different among the three modes. Other measures of ocular status, such as accommodative facility, relative accommodation, gradient AC/A, and phoria, exhibited no significant change between the pre- and postviewing data or among the three modes.

## 4. Discussion

In the present study, the observers watched 3D TV under three illumination modes. Before and after a 90-minute 3D viewing session under each mode, the ocular status, including accommodative facility, positive and negative relative accommodation, gradient AC/A, distance and near phoria, and positive and negative fusional vergence, was measured. Overall, we found that Mode B (the back illumination) and Mode C (the front illumination) produced the same effect on the different measures of ocular status; both modes were superior to Mode A (complete darkness) in terms of inducing less change after 3D viewing. Furthermore, Mode C produced the smallest change in accommodative microfluctuation. We conclude that the front illumination mode is much more appropriate for shutter 3D TV viewing when the change in ocular status is taken into account.

In previous research, Torii et al. [15] found that after viewing a 3D movie with film-patterned retarder glasses for 30 minutes, the amplitude of accommodation and vergence was decreased, while Fujikado [21] reported that subjects who viewed 3D video for 30 minutes experienced transient low-diopter myopia (0.2 D) due to excessive accommodation. Maeda et al. [6] found no significant difference between the accommodative microfluctuation data from before and after 90 or 60 minutes of 3D viewing in either the adult or child group; however, accommodative microfluctuation tended to increase after 3D viewing in some subjects. In the present study, we demonstrated that both the accommodative response and accommodative microfluctuation increased slightly after 3D viewing which was significantly under a certain mode of illumination (the accommodative response changed significantly under Mode A and the accommodative microfluctuation changed significantly under Mode A and Mode B). This result was consistent with the results of previous studies [6, 21]. The increased accommodative response and increased microfluctuation may occur when there is a convergence-accommodation conflict while viewing a 3D image. This conflict would increase accommodation and the burden on the accommodative system and result in incomplete relaxation of the visual system for a short time after viewing 3D TV [15].

As shown in Figure 7, the positive fusional vergence data of the viewers at a distance of 40 cm decreased significantly after 3D TV viewing under all three illumination modes, although no significant differences were found among these modes at a distance of 5 m. Previous studies have suggested that the change in reliance or fusional vergence could result in muscle and neural adaptation fatigue and lead to a series of visual symptoms [8, 16, 22, 23]. Wee et al. [24] found the increase of accommodative responses and the near point of convergence (decreased ability of convergence) after 30-minute 3D movies viewing with patterned retarder glasses, which did not occur after 30-minute 2D movies viewing. These results are very consistent with our study. In the present study, the distance and near phoria had a tendency to show more exophoria. Based on previous studies [25, 26], it would be logical to suggest that the decreased convergence and changed phoria after 3D TV viewing may be due to the adaptation of the vergence and accommodative controllers [27].

Apart from the above parameters (i.e., accommodative response and accommodative microfluctuation), the other measures of ocular status exhibited no significant differences after 3D viewing or among the three illumination modes. These no effect findings are somehow counterintuitive. As we know, the natural relationship between binocular convergence and accommodation is disrupted to perceive a single clear image while watching 3D TV, inducing more adaptation and better visual plasticity. However, the adaptation can disappear, and the disrupted cross-linking interaction between accommodation and vergence can recover after taking a break in 3D TV viewing [15, 23]. Torii et al. indicated that accommodative overshoot had lasting less than 1 min while viewing stereoscopic images [15], while Emoto et al. found that decreased fusional amplitude, after viewing of stereoscopic images, recovered to previewing levels in ten minutes [23]. The recovery time was indeterminate and depended on many factors. Once stopped exposing in the 3D viewing, the ocular status might recover in less than 20 minutes, which was the postmeasurement time of the present study. This recovery could be the reason for some of our current findings. Spatial frequency components, the disparity of the images, and long-term and frequent exposure to the 3D display as well as individual differences may account for the discrepancies among different studies [3].

Altogether, we arrived at the conclusion that ocular status is modulated not only by 3D viewing but also by the illumination mode. This finding may be attributed to the difference in luminance at the position of the viewer, the direction of the light source, or the familiar viewing environment of the participants. We did not examine the mechanism in this study, but we demonstrated that front illumination is an appropriate illumination mode for watching 3D displays. There is much evidence suggesting that illumination of the environment is a modifying factor forocular status [2, 3, 6], visual asthenopia [25, 28], and the contrast modulation of visual cortical cells [29, 30]. Low luminance levels, as in Mode A (complete darkness), would create inadequate viewing conditions and notable variations in the eyes when moving the gaze intermittently between the bright display and darker surroundings and would potentially cause eye strain. Increasing the ambient illumination to minimize differences in eye adaptation would potentially reduce visual fatigue for workers using typical LCDs in medical image soft-copy reading rooms [31]. Zhou et al. found that the selective reduction of monocular mean luminance in one eye can significantly affect the interocular balance in binocular combination in both normal and amblyopic observers [29]. Our results together with these previous reports suggest that the environmental illumination is critical in modulating our visual system and that a higher luminance such as that in Mode C produces a smaller change in ocular status.

We demonstrated that according to the type of illumination, 3D viewing can modify the ocular status with regard to visual plasticity to maintain a relatively stable visual system and thereby ensure that the cerebral cortex perceives a single clear image. The entire process is associated with the ocular muscles, nerves, or local brain areas that are involved in 3D viewing; therefore, the modification of visual plasticity may lead to the modification of these components of the visual system to a certain extent. We believe that this study may yield evidence of visual plasticity and provide clues for scientists working on neural plasticity.

## 5. Conclusion

We conclude that luminance plays a modulating role in ocular modification induced by the shutter display viewing of 3D images. Subjective accommodative function exhibits greater stability when illumination is in front of a viewer. Front illumination using fluorescent lamps may be an appropriate illumination mode for shutter 3D TV viewing.

## Figures and Tables

**Table 1:** Summary of the ocular status of observers before 3D TV viewing.

Number	Age (years)	Phoria (Δ)^*∗*^ (near)	Phoria (Δ)^*∗*^ (distance)	Refraction (spherical equivalent; diopter)
32	23.63 ± 1.58	−2.60 ± 5.05	−0.47 ± 2.21	−2.81 ± 1.43

^*∗*^A positive phoria is “eso” and a negative phoria is “exo.”

## References

[1] Suzuki Y., Onda Y., Katada S., Ino S., Ifukube T. (2004). Effects of an eyeglass-free 3-D display on the human visual system. *Japanese Journal of Ophthalmology*.

[2] Kim S.-H., Suh Y.-W., Yun C.-M., Yoo E.-J., Yeom J.-H., Cho Y. A. (2013). 3D asthenopia in horizontal deviation. *Current Eye Research*.

[3] Fukushima T., Torii M., Ukai K., Wolffsohn J. S., Gilmartin B. (2009). The relationship between CA/C ratio and individual differences in dynamic accommodative responses while viewing stereoscopic images. *Journal of Vision*.

[4] Nishina S., Wakayama A., Miki A. (2013). [Viewing 3D stereoscopic images in children and adults with and without strabismus: multicenter study in Japan]. *Nippon Ganka Gakkai zasshi*.

[5] Lin Y., Fotios S., Wei M., Liu Y., Guo W., Sun Y. (2015). Eye movement and pupil size constriction under discomfort glare. *Investigative Ophthalmology and Visual Science*.

[6] Maeda F., Tabuchi A., Kani K., Kawamoto K.-I., Yoneda T., Yamashita T. (2011). Influence of three-dimensional image viewing on visual function. *Japanese Journal of Ophthalmology*.

[7] Sheedy J. E., Hayes J., Engle J. (2003). Is all asthenopia the same?. *Optometry and Vision Science*.

[8] Jones R., Stephens G. L. (1989). Horizontal fusional amplitudes. Evidence for disparity tuning. *Investigative Ophthalmology & Visual Science*.

[9] Michaelides M., Hardcastle A. J., Hunt D. M., Moore A. T. (2006). Progressive cone and cone-rod dystrophies: phenotypes and underlying molecular genetic basis. *Survey of Ophthalmology*.

[10] Yao Y.-J., Chang Y.-M., Xie X.-P., Cao X.-S., Sun X.-Q., Wu Y.-H. (2008). Heart rate and respiration responses to real traffic pattern flight. *Applied Psychophysiology Biofeedback*.

[11] Carozzi G. (1983). Illumination of the work environment. *Rivista Italiana Degli Odontotecnici*.

[12] Lin C. J., Feng W.-Y., Chao C.-J., Tseng F.-Y. (2008). Effects of VDT workstation lighting conditions on operator visual workload. *Industrial Health*.

[13] Taptagaporn S., Sotoyama M., Saito S., Suzuki T., Saito S. (1995). Visual comfort in VDT workstation design. *Journal of Human Ergology*.

[14] Shieh K.-K., Lai Y.-R. (2008). Effects of ambient illumination, luminance contrast, and stimulus type on subjective preference of vdt target and background color combinations. *Perceptual and Motor Skills*.

[15] Torii M., Okada Y., Ukai K., Wolffsohn J. S., Gilmartin B. (2008). Dynamic measurement of accommodative responses while viewing stereoscopic images. *Journal of Modern Optics*.

[16] Zhang L., Zhang Y.-Q., Zhang J.-S., Xu L., Jonas J. B. (2013). Visual fatigue and discomfort after stereoscopic display viewing. *Acta Ophthalmologica*.

[17] Gray L. S., Winn B., Gilmartin B. (1993). Effect of target luminance on microfluctuations of accommodation. *Ophthalmic and Physiological Optics*.

[18] Day M., Seidel D., Gray L. S., Strang N. C. (2009). The effect of modulating ocular depth of focus upon accommodation microfluctuations in myopic and emmetropic subjects. *Vision Research*.

[19] Day M., Gray L. S., Seidel D., Strang N. C. (2009). The relationship between object spatial profile and accommodation microfluctuations in emmetropes and myopes. *Journal of Vision*.

[20] Sheppard A. L., Davies L. N. (2010). Clinical evaluation of the Grand Seiko Auto Ref/Keratometer WAM-5500. *Ophthalmic and Physiological Optics*.

[21] Fujikado T. (1997). Asthenopia from the viewpoint of visual information processing-effect of watching 3D images. *Journal of the Eye*.

[22] Emoto M., Niida T., Okano F. (2005). Repeated vergence adaptation causes the decline of visual functions in watching stereoscopic television. *Journal of Display Technology*.

[23] Emoto M., Nojiri Y., Okano F. (2004). Changes in fusional vergence limit and its hysteresis after viewing stereoscopic TV. *Displays*.

[24] Wee S. W., Moon N. J., Lee W. K., Jeon S. (2012). Ophthalmological factors influencing visual asthenopia as a result of viewing 3D displays. *British Journal of Ophthalmology*.

[25] Karpicka E., Howarth P. A. (2013). Heterophoria adaptation during the viewing of 3D stereoscopic stimuli. *Ophthalmic and Physiological Optics*.

[26] Sreenivasan V., Bobier W. R., Irving E., Lakshminarayanan V. (2010). Effect of vergence adaptation on convergence accommodation: model simulations. *IEEE Transactions on Bio-Medical Engineering*.

[27] Costa Lança C., Rowe F. J. (2016). Variability of fusion vergence measurements in heterophoria. *Strabismus*.

[28] Howarth P. A. (2011). Potential hazards of viewing 3-D stereoscopic television, cinema and computer games: a review. *Ophthalmic and Physiological Optics*.

[29] Zhou J., Jia W., Huang C.-B., Hess R. F. (2013). The effect of unilateral mean luminance on binocular combination in normal and amblyopic vision. *Scientific Reports*.

[30] Zhou J., Liu R., Feng L., Zhou Y., Hess R. F. (2016). Deficient binocular combination of second-order stimuli in amblyopia. *Investigative Ophthalmology and Visual Science*.

[31] Chawla A. S., Samei E. (2007). Ambient illumination revisited: a new adaptation-based approach for optimizing medical imaging reading environments. *Medical Physics*.

